# Special Issue “Characteristics of Dental Ceramics”

**DOI:** 10.3390/ma19071363

**Published:** 2026-03-30

**Authors:** Marina Amaral, Kusai Baroudi

**Affiliations:** 1 Department of Dentistry, University of Taubaté, Taubaté 12020-270, Brazil; marina.amaral@unitau.br; 2 Department of Clinical Sciences, College of Dentistry, Ajman University, Ajman P.O. Box 346, United Arab Emirates

Dental ceramics have become indispensable materials in contemporary restorative and prosthetic dentistry. The continuous evolution of clinical workflows, digital manufacturing systems, and patient-driven demands for highly esthetic and durable restorations have led to increased scientific and clinical interest in ceramic materials. In this context, understanding the characteristics of dental ceramics remains crucial for improving clinical outcomes and ensuring long-term treatment success. This Special Issue, “Characteristics of Dental Ceramics,” was conceived to advance knowledge in this rapidly evolving field and to consolidate the current scientific evidence regarding the performance and applications of ceramic materials in dentistry.

Over the past decade, remarkable progress has been observed in ceramic composition, processing techniques, and restorative protocols. Glass ceramics, lithium silicate derivatives, and polycrystalline ceramics, particularly zirconia-based systems, have expanded their indications from single-unit restorations to complex rehabilitations, including implant-supported prostheses and minimally invasive restorations. Meanwhile, CAD/CAM technologies have optimized manufacturing precision and clinical efficiency, enabling restorations with improved structural reliability [1].

However, despite these advances, important challenges persist. Mechanical complications such as debonding, chipping, fatigue-related fractures, and wear interactions with antagonist dentition remain clinically relevant concerns [2,3,4]. Furthermore, the growing availability of high-translucency zirconia and hybrid ceramic systems has introduced new questions regarding the balance between esthetics and mechanical performance, particularly under long-term cyclic loading conditions. Variability in laboratory testing protocols and inconsistencies between in vitro findings and clinical outcomes continue to limit the translation of experimental results into predictable clinical recommendations [5].

Another knowledge gap pertains to the interaction between ceramic materials and contemporary adhesive and cementation strategies [6,7]. Although adhesive luting has enhanced restoration retention and load distribution, clinical failures associated with inadequate bonding protocols, aging degradation, and hydrolytic effects remain insufficiently understood. Additionally, the long-term effects of surface treatments, glazing, polishing procedures, and clinical adjustments on wear behavior and fatigue resistance require further investigation [8,9].

Biocompatibility and optical stability also remain critical topics, especially considering the increasing demand for highly esthetic restorations in anterior regions. Color alteration over time, surface degradation in oral environments, and the influence of material thickness and translucency on final restoration appearance continue to challenge clinicians and researchers alike [10]. The integration of digital shade selection, characterization techniques, and optical simulation tools highlights the need for interdisciplinary approaches combining material science and clinical dentistry.

The aim of this Special Issue was to address several of these knowledge gaps by gathering contributions that explore ceramic materials from multiple scientific and clinical perspectives. Collectively, these contributions reinforce the importance of linking laboratory evidence with clinical applicability, thereby supporting more predictable and durable restorative solutions.

Nevertheless, future research directions must continue to evolve alongside clinical demands and technological innovations. One major focus should involve long-term clinical trials comparing new ceramic generations with established materials, allowing evidence-based decision-making regarding material selection and indication limits. Additionally, research integrating digital workflows with predictive mechanical modeling may support restoration design optimization tailored to individual patient risk profiles.

Another promising avenue involves the incorporation of artificial intelligence and machine learning tools for predicting restoration survival based on patient-specific parameters, occlusal conditions, and material properties. Multi-scale analyses connecting microstructural features to macroscopic mechanical behavior may further improve material development and customization. Sustainability considerations and environmentally responsible manufacturing processes also represent emerging areas of interest in dental material science.

Finally, strengthening collaboration between material scientists, dental clinicians, and digital technology developers will be essential to accelerate innovation while ensuring clinical reliability. Future ceramic systems must simultaneously achieve esthetic excellence, mechanical resilience, simplified clinical protocols, and long-term biological compatibility.
